# Current approaches for automated model building into cryo-EM maps using *Buccaneer* with *CCP-EM*

**DOI:** 10.1107/S2059798320005513

**Published:** 2020-05-29

**Authors:** Soon Wen Hoh, Tom Burnley, Kevin Cowtan

**Affiliations:** a York Structural Biology Laboratory, Department of Chemistry, University of York, York YO10 5DD, United Kingdom; b Scientific Computing Department, Science and Technology Facilities Council, Research Complex at Harwell, Didcot OX11 0FA, United Kingdom

**Keywords:** model building, *Buccaneer*, cryo-EM, Collaborative Computational Project for Electron cryo-Microscopy, *CCP-EM*

## Abstract

This work focuses on the use of the existing protein-model-building software *Buccaneer* to provide structural interpretation of electron cryo-microscopy (cryo-EM) maps. Originally developed for application to X-ray crystallography, the necessary steps to optimise the usage of *Buccaneer* with cryo-EM maps are shown. This approach has been applied to the data sets of 208 cryo-EM maps with resolutions of better than 4 Å. The results obtained also show an evident improvement in the sequencing step when the initial reference map and model used for crystallographic cases are replaced by a cryo-EM reference. All other necessary changes to settings in *Buccaneer* are implemented in the model-building pipeline from within the *CCP-EM* interface (as of version 1.4.0).

## Abstract 2

The model-building software *Buccaneer* has been repurposed based on existing methods for building models into cryo-EM maps. These approaches are implemented in the latest *CCP-EM* model-building pipeline (version 1.4.0).

## 1. Introduction   

Technological advances have brought major improvements in single-particle electron cryo-microscopy (cryo-EM), in particular the introduction of direct electron detectors (McMullan *et al.*, 2016 ▸). This technique has allowed the study of rapidly frozen biological macromolecules without the need for crystallization. The increasing number of cryo-EM maps produced in recent years, especially those of high resolution (<4 Å), has motivated the modification of existing and the development of new model-building tools to interpret cryo-EM maps (Cowtan, 2008 ▸; Baker *et al.*, 2012 ▸; Brown *et al.*, 2015 ▸; Wang *et al.*, 2015 ▸; Chen *et al.*, 2016 ▸; Zhou *et al.*, 2017 ▸; Terwilliger, Adams *et al.*, 2018 ▸; Terashi & Kihara, 2018 ▸; Afonine, Poon *et al.*, 2018 ▸; Chojnowski *et al.*, 2018 ▸; Nicholls *et al.*, 2018 ▸).

The interpretation of cryo-EM maps is slightly different from that of X-ray crystallographic maps since there is no phase problem as in crystallography. In the X-ray case, phases must be recovered either by fitting a related molecule into the crystal lattice or by experimental techniques. Density-modification, iterative model-building and refinement procedures are then performed to improve the initial maps and phases (Cowtan & Zhang, 1999 ▸; Terwilliger, 2000 ▸, 2002 ▸; Terwilliger *et al.*, 2008 ▸; Cowtan, 2010 ▸). In cryo-EM, volumes produced using the single-particle analysis reconstruction technique contain both amplitude and phase information. However, in these EM cases the amplitudes are less accurate than those measured from X-ray diffraction (Cheng, 2015 ▸). In addition, heterogeneity of the sample and some degree of error in the reconstruction step will cause blurring of signal in the map (Rosenthal & Henderson, 2003 ▸). Thus, sharpening/blurring of a cryo-EM map is necessary for optimal interpretation. Sharpening/blurring is a procedure that is performed after the reconstruction step but before model building. Different parts of the map may require different levels of sharpening or blurring, although a single global sharpening/blurring parameter can be sufficient to produce a map for visualisation and model building (Jakobi *et al.*, 2017 ▸; Terwilliger, Sobolev *et al.*, 2018 ▸). Although modifications of amplitudes via sharpening can be performed prior to model building to increase the interpretability of input maps, phases are not modified during refinement of the model. We note that the experimental phases are not without error, and it is hoped that in the future a model-based error model (or similar) could be used to improve both the amplitudes and the phases of the data collected.

It should be noted that the maps produced by cryo-EM represent Coulomb or electric potential as opposed to the electron density derived from X-ray crystallography. However, using appropriately modified scattering factors allows the use of the existing Fourier-based techniques that were developed for the analysis of X-ray data. Nevertheless, there are qualitative differences that need to be accounted for (Nicholls *et al.*, 2018 ▸) and appropriate adjustments have to be made in order for the existing tools to work well with cryo-EM data. In this work, we have repurposed *Buccaneer* to work with EM data. We report the approach taken and its results.

## 2. Method   

### 2.1. Overview of the automated model-building pipeline in *CCP-EM*   

**Figure 1:**
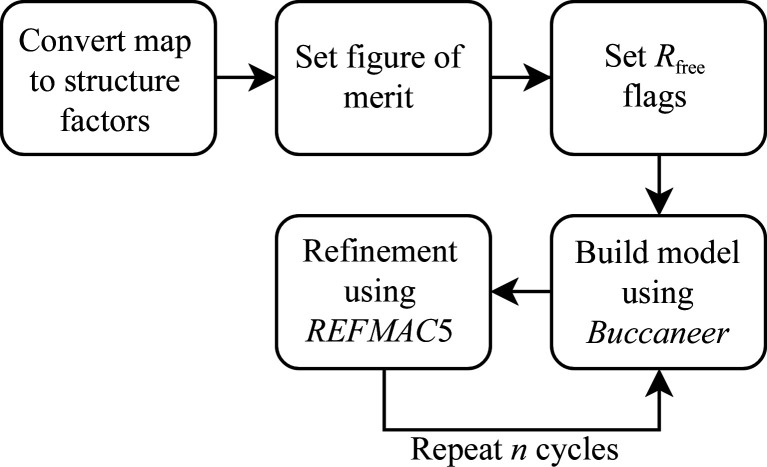
Overview of the automated model-building pipeline using *Buccaneer* in the *CCP-EM* software suite.

The automated model-building pipeline within the *CCP-EM* interface (Burnley *et al.*, 2017 ▸) consists of several subtasks, as shown in Fig. 1 ▸. The inputs required are a map file in MRC (also known as CCP4) format, a sequence file (in FASTA format) containing the sequences of the unique chains of the target structure, an estimate of the resolution of the map and a global sharpening parameter (Collaborative Computational Project for Electron cryo-Microscopy, 2019 ▸). By default, no sharpening of the map is performed if the global sharpening parameter is left at 0. An initial model can optionally be provided for model extension or completion. A range of other optional parameters which affect the behaviour of the *Buccaneer* (Cowtan, 2019 ▸) or *REFMAC*5 (Murshudov, 2019 ▸) software can be provided in the designated keyword section of the user interface.

Firstly, structure factors are calculated from the input map given using *REFMAC*5 (Nicholls *et al.*, 2018 ▸). Higher resolution data are truncated to the specified resolution and the global sharpening factor (if specified) is also applied in the calculation. An output file is written in MTZ format. Standard deviation of structure amplitude (SIGFP) and figure of merit for the experimental phases (FOM) dummy columns are added to the MTZ file with their values set to 1. This task is performed using *SFTOOLS*. *FREERFLAG* is then used to tag each reflection in the MTZ file with a flag (this is for software compatibility and is not used as a quality metric).

Secondly, iterative model-building and refinement tasks are performed using *Buccaneer* and *REFMAC*5, respectively. The number of model-building and refinement iterations are set to five cycles and can be changed easily by users as required. One full cycle is defined as a continuous procedure of model building followed by structure refinement. The refined structure at the end of each cycle is used as an initial model in the next cycle of model building. *Buccaneer* uses a common form of likelihood function with different parameterizations to trace the protein main chain and sequence the residues. Brown *et al.* (2015 ▸) have implemented an EM mode in *REFMAC*5 to facilitate the refinement of structures solved by cryo-EM. The keyword ‘source EM MB’ allows the use of form factors described by the relationship between X-ray and electron scattering form factors via the Mott–Bethe formula (Kirkland, 2010 ▸; Murshudov, 2016 ▸). As mentioned previously, there is no phase problem in cryo-EM, so standard crystallographic density-modification techniques should not be used for phase ‘improvement’ (Murshudov, 2016 ▸). In subsequent pipeline cycles, the initially generated structure factors are used again for model building and refinement.

At the end of the pipeline, autobuilding and refinement statistics are shown in the results tab. This information from each cycle of *Buccaneer* and *REFMAC*5 is shown in table and graph forms.

### 2.2. Adaptations for building into cryo-EM maps   

#### 2.2.1. Cryo-EM known reference map and model   

One approach taken to improve the performance of *Buccaneer* with cryo-EM maps is to replace the known X-ray reference map and model, which are used by the feature-recognition code to infer how protein motifs are likely to appear at a given resolution, with an EM reference structure. This is used in the application of *FFFear* target and weight functions to locate, extend and sequence the protein chain, as explained thoroughly by Cowtan (2008 ▸). In brief, the calculated mean and variance of the electron density for all occurrences of the search target in the known reference structure at a similar resolution to the unknown structure are used to construct the likelihood target and weight functions (Cowtan, 2006 ▸, 2008 ▸). A search target based on a 4 Å sphere centred on the C^α^ atom is used to locate probable C^α^-atom positions. Targets based on a 5.5 Å sphere of density centred on the C^β^ atom are used to classify side-chain types. We used the reconstructed map of β-galactosidase (EMD-4116; Kimanius *et al.*, 2016 ▸) and fitted the previously released structure (PDB entry 5a1a) for EMPIAR entry 10061 (Bartesaghi *et al.*, 2015 ▸) as new reference data in *Buccaneer* for cryo-EM cases, which was chosen on the basis of size, data resolution and performance in model building on EM maps (see Section 3).

#### 2.2.2. Fast and correlation modes in *Buccaneer*   

The *Buccaneer* software features a version of the the target function that corrects the scale and offset of the map, which is activated by the keyword ‘correlation-mode’. This ensures the effective location of the search fragment in a map by placing the features in the fragment density on the same scale and with the same offset as those in the map. This is important because EM maps are not typically scaled to the absolute V/Å scale, and internal scaling of the map in *Buccaneer* proved to be ineffective.

Correlation mode is not implemented for the initial search for chain ‘seed’ positions, which makes use of fast Fourier transforms. An alternative search procedure, activated by the keyword ‘fast’, searches for very short helix-like and strand-like features using a scoring function which is independent of the scale of the density. While originally developed as a faster alternative to the Fourier search, it is employed here for its scale-invariance property.

## 3. Results and discussion   

Unless specified otherwise, all tests were performed on a total of 208 EM maps downloaded from the Electron Microscopy Data Bank (EMDB; Lawson *et al.*, 2011 ▸). The published resolutions of the maps were in the range 1.8–3.97 Å. Map searches were performed on three separate occasions. Criteria such as single particle, protein and a resolution of better than 4.0 Å were used to filter the search results. Maps were excluded if no deposited structure was available at the time of the search and if the volume was too large (for example maps of virus capsids). An initial test was performed to examine the effects of correlation and fast modes on models built by *Buccaneer*. Results were obtained from one cycle of *Buccaneer* performed in four different settings without applying any sharpening to the map.

(1) Turning on both correlation and fast modes.
(2) Turning on only correlation mode.
(3) Turning on only fast mode.
(4) Turning off both correlation and fast modes.

**Figure 2:**
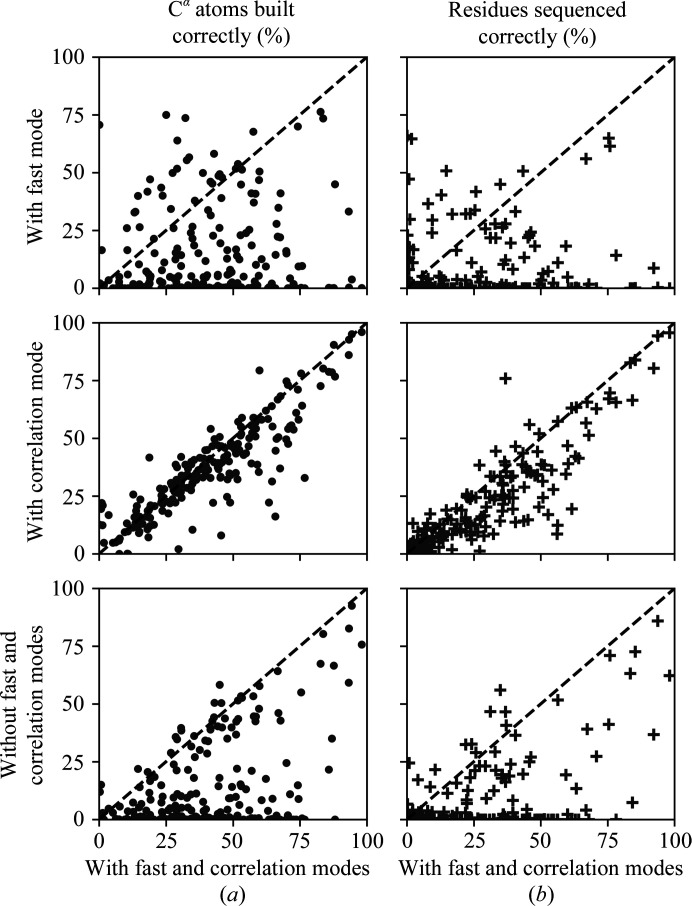
Comparison of percentages of (*a*) C^α^ atoms built (filled circles) and (*b*) residues sequenced (plus symbols) correctly in one cycle of *Buccaneer* applying various combinations of correlation and fast modes using EM reference data. Markers above and below the diagonal dashed line correspond to better results with the respective modes turned on and off, respectively. Top: results with fast mode on versus both modes on. Middle: results with correlation mode on versus both modes on. Bottom: results with both modes off versus both modes on.

The effects of correlation and fast modes on C^α^ atoms built and sequenced are visualized as comparison scatter plots in Fig. 2 ▸. The mean values of correct C^α^ atoms built and residues sequenced for 208 models are tabulated in Table 1 ▸. The mean differences in the number of correct C^α^ atoms built between options 1 and 2, options 1 and 3, and options 1 and 4 are 25.4%, 3.7% and 25.8%, respectively. The mean differences in the number of residues sequenced correctly between the combinations 1–2, 1–3 and 1–4 are 16.1%, 5.2% and 16.2%, respectively. A majority of the maps have a higher percentage of residues built and sequenced correctly when both correlation and fast modes are turned on in *Buccaneer*, as seen in Fig. 2 ▸, where most of the markers are below the diagonal dashed line in the plots (refer to Supplementary Table S1 for the numerical details). These results demonstrate that for optimum performance both correlation and fast modes should be turned on in *Buccaneer* for model building into cryo-EM maps.

In X-ray cases, fast mode is always turned on to activate a faster alternative to the Fourier search. Correlation mode is always on from the first cycle of the model-building pipeline when a molecular-replacement solution is used. On the other hand, when building into an empty map from experimental phasing correlation mode is turned off in the first cycle.

**Figure 3:**
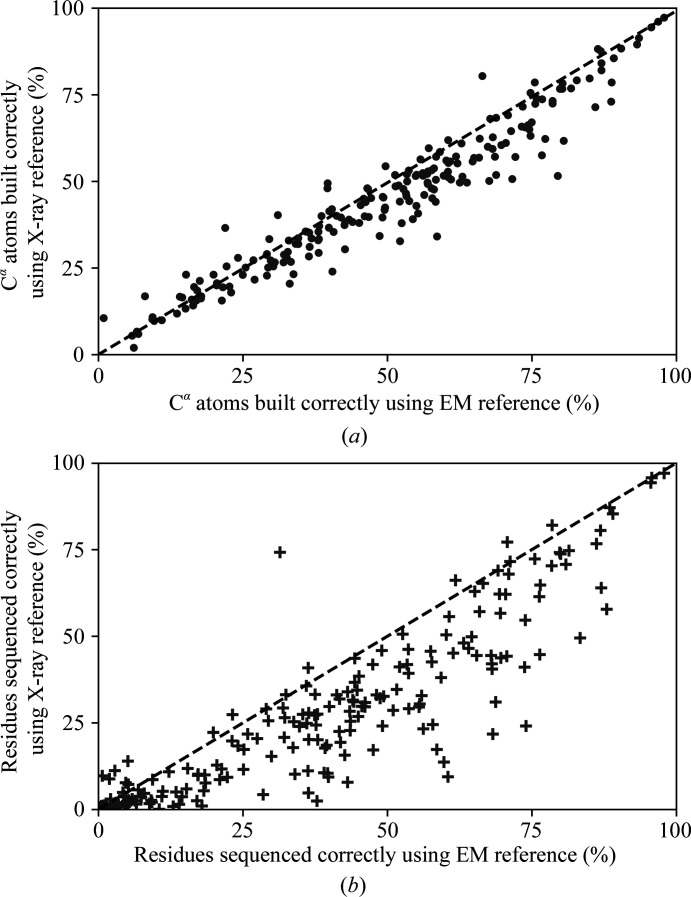
Comparison of percentages of (*a*) C^α^ atoms built (filled circles) and (*b*) residues sequenced (plus symbols) by *Buccaneer* using X-ray versus EM reference data. Markers above and below the diagonal dashed line correspond to better results from using X-ray and EM reference data, respectively.

Next, the automated model-building pipeline procedure is performed on the EM maps, with a total of 25 cycles each without applying any sharpening. Models from the best cycle are compared with the respective published fitted model for each map in terms of model completion. Model completion is evaluated using the number of correctly positioned C^α^ atoms within 1 Å of the corresponding C^α^ atoms in the fitted model and the number of correctly sequenced residues. The best cycle is selected based on the average Fourier shell correlation (FSC_average_) value reported at the end of refinement by *REFMAC*5, where a higher value indicates a better fit to density (Brown *et al.*, 2015 ▸). Comparisons of the number of C^α^ atoms built and residues sequenced correctly for the best models are shown in Fig. 3 ▸. An indication of the overall and local fit of the deposited models to maps are defined by the global cross-correlation coefficient (CCC) and Segment-based Manders’ Overlap Coefficient, respectively, in *TEMPy* (Farabella *et al.*, 2015 ▸; Joseph *et al.*, 2017 ▸). Similar map–model correlation coefficients are also calculated using the model and data fit validation tool in *Phenix* (Afonine, Klaholz *et al.*, 2018 ▸). These model-to-map fitness scores are tabulated in Supplementary Table S2.

The use of a cryo-EM reference structure in *Buccaneer* produced better results when compared with using an X-ray reference structure. A significant improvement is seen in the sequencing step of the software, where the majority of the 180 cases improve, as shown in Fig. 3 ▸(*b*). Of the 180 cases, 41 achieved an improvement of greater than 20% (see Table 2 ▸). However, the improvement in the C^α^ finding step is not as great as that in the sequencing step, as seen in Fig. 3 ▸(*a*), with the markers clustering nearer to the diagonal dashed line (169 cases). Three of these 169 cases were observed to have a greater than 20% improvement in the C^α^ finding step (see Table 2 ▸). The sequencing algorithm in *Buccaneer* is based on a machine-learning approach, and parameterization of the likelihood function does play a role in determining its effectiveness. As such, it is not surprising that using a cryo-EM reference structure improves the performance, as this more closely represents the target map and cryo-EM maps have systemic differences compared with those derived from X-ray diffraction. X-ray photons are scattered by electrons, but electrons are scattered by Coulomb interaction; therefore, different maps are expected for X-ray diffraction versus EM. In addition, electrostatic potential maps can include negative features from negatively charged atoms (Yonekura & Maki-Yonekura, 2016 ▸). The presence of multiple repeated subunits, which is seen in three cases (EMD-3838, EMD-6830 and EMD-8162), will result in multiplication of the correct number of residues built and sequenced. Since the statistics are calculated based on the total number of residues present and the fitted model and not by monomeric unit, a higher improvement percentage is observed for these three cases. A summary list of entries along with the PDB code, resolution and comparison statistics of the respective published models has been tabulated (Supplementary Table S3).

From the results obtained using an EM reference structure, we observed 30 cases with built models with a completeness of greater than or equal to 75% in terms of correct C^α^ atoms built (Table 3 ▸). In terms of residues sequenced correctly, only 20 cases achieved a model completeness of 75%. Just under half of the test set have models with less than 50% completeness in terms of C^α^ atoms built. However, more than half of the test cases have built models with less than 50% completeness in sequence.

For results obtained using an X-ray reference structure, the number of built models with greater than or equal to 75% completeness in terms of correct residues built and sequenced are 21 and nine, respectively. More than half of the structures built have less than 50% model completeness.

**Figure 4:**
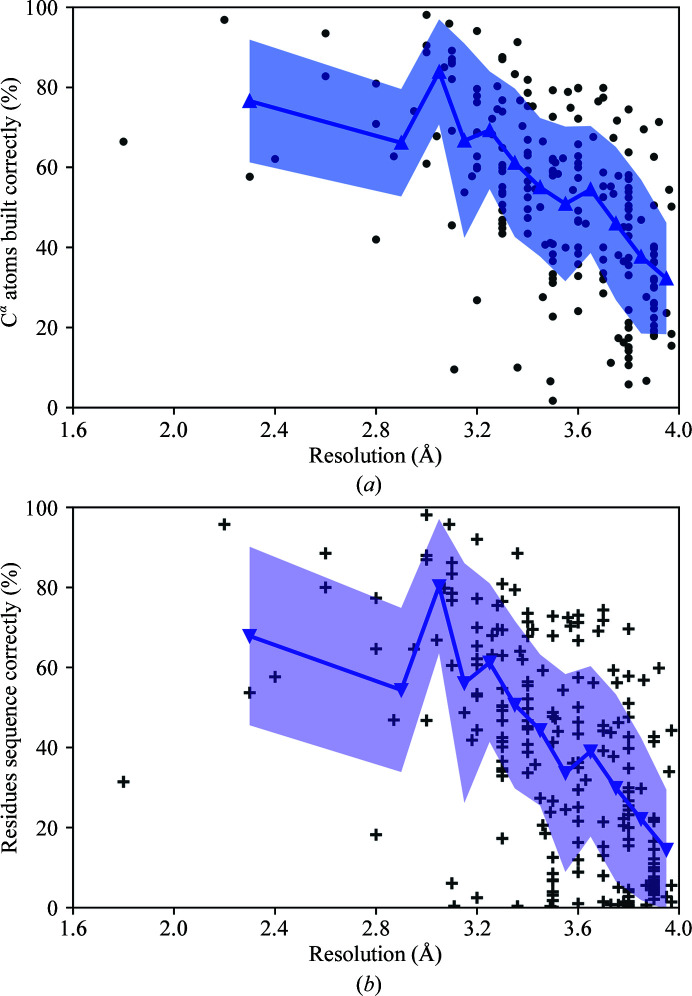
Percentage of (*a*) C^α^ atoms built and (*b*) residues sequenced by *Buccaneer* across resolutions of 1.8–3.97 Å. Lines with markers show the means for the resolution bins 1.8–2.8 Å and 2.8–3.0 Å and for resolution bins of 0.1 Å in size from 3.0 to 4.0 Å. Areas filled with colour indicate the standard deviation of values within each bin.

The percentages of correct C^α^ atoms built and residues sequenced across resolution bins from 1.5 to 4 Å are shown in Fig. 4 ▸. *Buccaneer* is able to interpret EM maps with variable efficiencies across the range of resolutions. A noticeable decrease in model completeness is observed as the map resolution becomes worse. However, the completeness of the model built varies with different maps at the same reported resolutions. For example, the percentage of C^α^ atoms built correctly in maps with resolutions between 3.3 and 3.4 Å can be as high as 91% and as low as 10%. Further investigation is needed to determine the causes of such variability.

**Figure 5:**
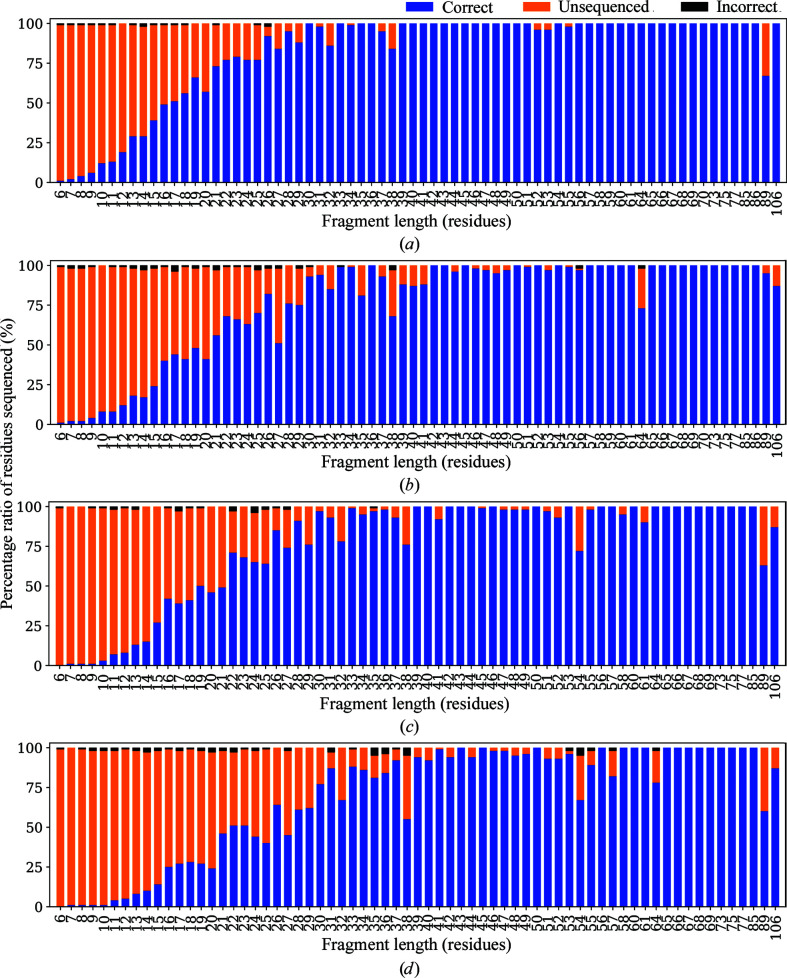
Average success rates in sequencing fragments of different lengths for 109 models using two different chain traces and reference structures. (*a*) Fragments cut from the deposited models sequenced using an EM reference structure. (*b*) Fragments cut from the deposited models sequenced using an X-ray reference structure. (*c*) Fragments from the *Buccaneer* models sequenced using an EM reference structure. (*d*) Fragments from the *Buccaneer* models sequenced using an X-ray reference structure.

We have also evaluated the success rates in sequencing fragments of different lengths. A large set of chain fragments with different lengths from the starting models were used in this evaluation. Two starting models were used for each case: firstly the model constructed by running one cycle of *Buccaneer* using the X-ray reference structure data and secondly the model deposited in the EMDB. Residues and segments from the constructed model which were incorrectly traced were removed, since they cannot be sequenced. Some initial models constructed by *Buccaneer* were excluded from this evaluation after removing the incorrectly traced residues since they resulted in fragments of five residues or fewer in length. The deposited model was chopped into different lengths based on the constructed model, where displacements between corresponding C^α^ atoms are within 1 Å. Sequencing of the fragments was performed using the EM and X-ray references in separate tests. Results are classified as correctly sequenced, incorrectly sequenced and not sequenced residues. The proportions of these classes are plotted in Fig. 5 ▸. Owing to the method by which fragments were obtained, longer fragments are small in number, so results from fragments with 30 residues and above show more errors and some statistical noise.

Overall, there is little difference between using a chain traced from the refined deposited model and a *Buccaneer* model as a starting point for sequencing. Noticeable differences can be seen when comparing the results obtained using different reference structure data, with chains being better sequenced with the use of an EM reference. When using the deposited model with the EM reference, fragments starting from 17 residues achieve a 50% success rate, while an 80% success rate is achieved with fragments of 26 residues or more. With the X-ray reference, fragments with at least 21 residues are required to achieve a 50% success rate and at least 30 residues are needed for an 80% success rate.

For the autobuilt model, a success rate of 50% is achieved with fragments of 19 and 21 residues using EM and X-ray references, respectively. A success rate of 80% requires fragments with 26 residues or more using an EM reference, while with an X-ray reference at least 31 residues are required to achieve an 80% success rate. Wrongly sequenced fragments are reduced with the use of an EM reference, but are still rare in all cases.

**Figure 6:**
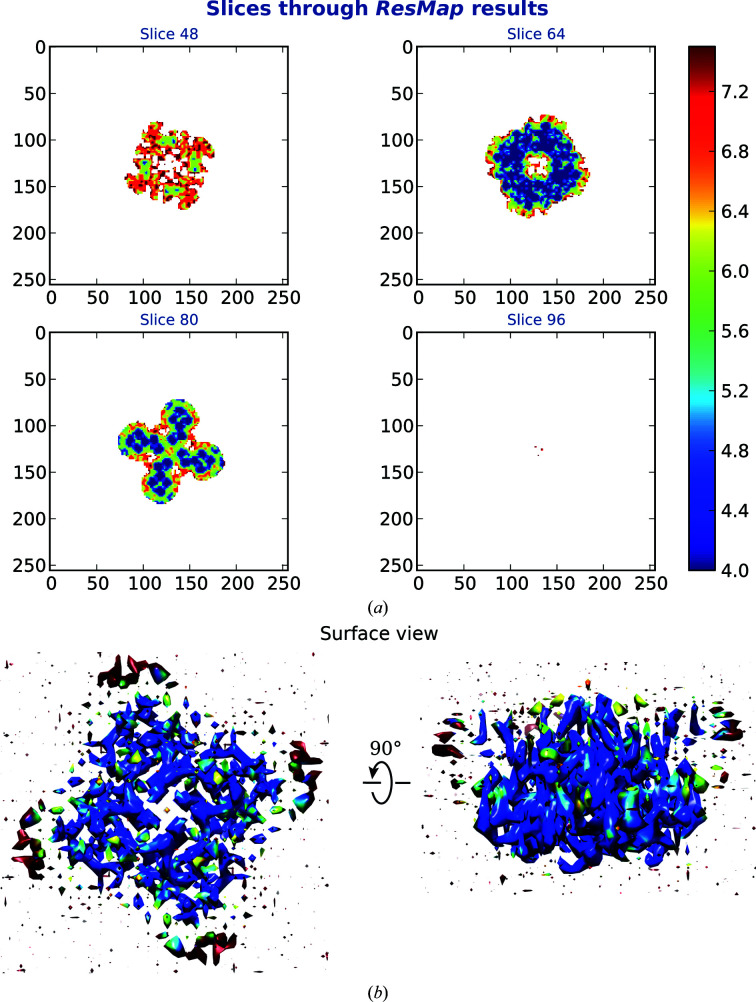
Estimated local resolution for the map from EMD-8912 calculated using *ResMap*. The surface view was generated using *UCSF Chimera* (Pettersen *et al.*, 2004 ▸) with a 0.06 contour level.

One reason for unsuccessful autobuilding or sequencing of a model is owing to poorly resolved regions in a map. This can be seen in one of the test data sets (EMD-8912) of a polycystic kidney disease 2-like 1 (polycystin 2-l1) ion channel (Hulse *et al.*, 2018 ▸). The best model produced from the automated model-building pipeline achieved a build completeness of 9%, and only 0.5% of the residues were sequenced correctly. The stated resolution of the map, reportedly 3.11 Å in the EMDB or 3.3 Å in the article, does not seem to relate to the quality of the map. The authors and peer reviewers of the article also stated that the low resolution hindered the building of side chains in the model. It is worth noting that the overall resolution reported for cryo-EM maps is the predefined cutoff of the FSC curve calculated between two half maps (Rosenthal & Henderson, 2003 ▸). This tells us the global reliability of the information content. However, regions that are flexible or have varying occupancy will have resolutions that differ from the reported overall resolution. We used *ResMap* (Kucukelbir *et al.*, 2014 ▸) to determine the local resolution of the map, and plots are shown in Fig. 6 ▸. The mean resolution estimated by *ResMap* for this map is 5.54 Å, with the majority of the voxels falling within 4–6 Å. There are maps of a similar system, polycystin 2, within the data set that we have used. These are EMD-8354 (Shen *et al.*, 2016 ▸), EMD-6877 (Su *et al.*, 2018 ▸) and EMD-7786 (Zheng *et al.*, 2018 ▸), with maps of resolutions 3, 3.38 and 3.54 Å, respectively. The autobuilt models produced for EMD-8354, EMD-6877 and EMD-7786 achieved build completenesses of 90%, 69% and 61%, respectively. The completenesses in the sequence of models for EMD-8354, EMD-6877 and EMD-7786 were 87%, 60% and 54%, respectively (see Supplementary Table S3).

**Figure 7:**
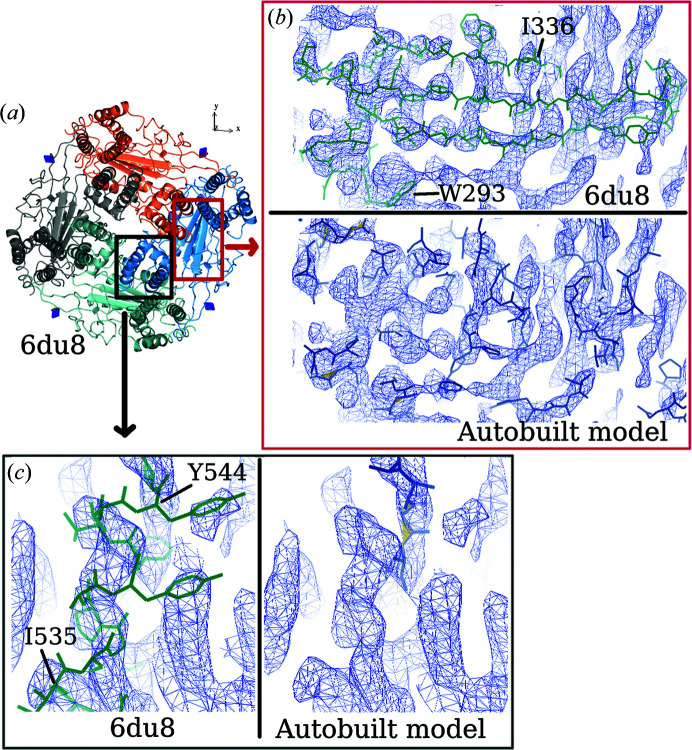
Comparing parts of the deposited model (PDB entry 6du8) and the autobuilt model built into a map from EMD-8912. (*a*) Overall view of the deposited model. Close-up views are shown of (*b*) a β-strand region and (*c*) a helix part from the map. The contour level used is 0.14.

**Figure 8:**
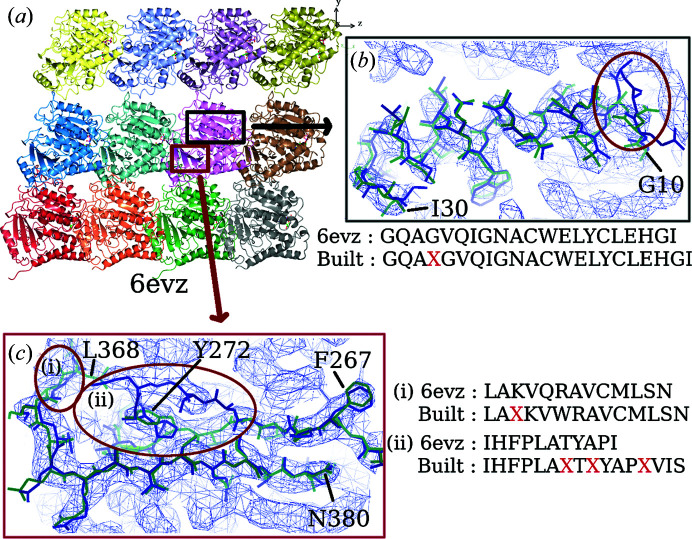
Comparing the deposited model (PDB entry 6evz) and the autobuilt model built into a map from EMD-3964. (*a*) Overall view of the deposited model. Close-up views are shown of (*b*) a helix chain and (*c*) a β-strand region from the map. The contour level used is 0.16. Circled regions show extra residues built incorrectly into the chain. The red X in the sequence indicates the location of an extra built residue.

The quality of the local region of the map often determines the completeness of an autobuilt model. Fig. 7 ▸ shows close-up views of a β-strand region and a helix chain of the map from EMD-8912. The volumes are disconnected where the main chains are located and side-chain features are not distinct. This caused the incorrect connection of the residues built (Fig. 7 ▸ *b*). Maps in which features of secondary structure can be observed often yield autobuilt models with higher completeness. Figs. 8 ▸ and 9 ▸ show parts of the maps from EMD-3964 and EMD-8354, respectively, where users can expect an accurately built and sequenced model from the pipeline. Maps from both EMD-3964 and EMD-8354 display rather sharp secondary-structure features. Side-chain features are also more distinct when compared with the map from entry EMD-8912. Fig. 8 ▸ displays situations such as incorrectly linked main-chain residues or extra residues built which can occur during model building in *Buccaneer*.

**Figure 10:**
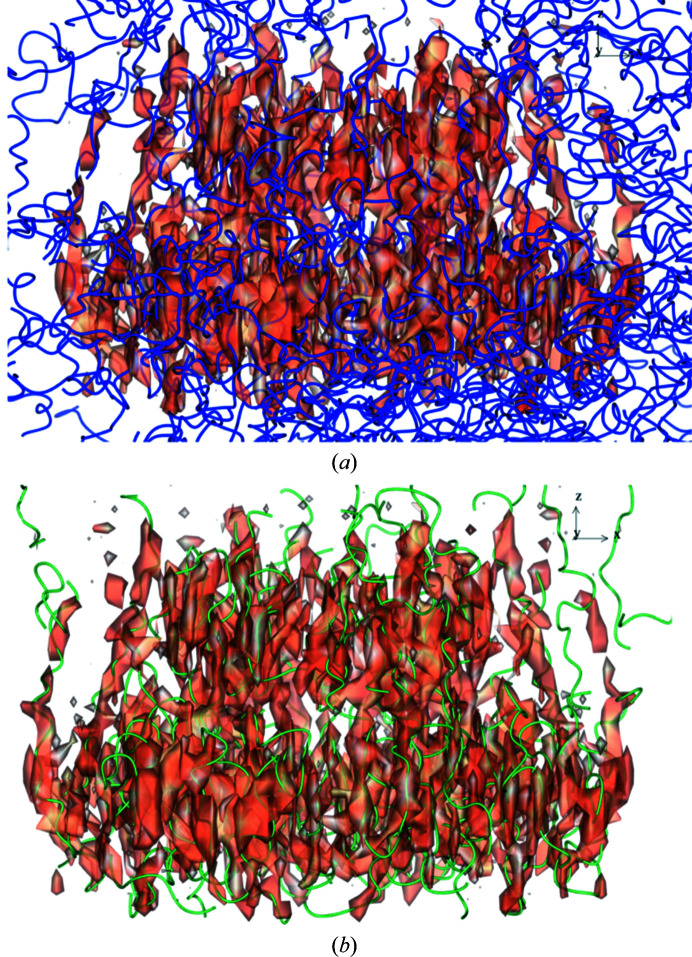
A view of the amount of fragments built in a noisy map. Autobuilt models are shown for EMD-8912 built on (*a*) an unmasked map and (*b*) a masked map. Models are represented as C^α^ backbone strands. The map surface view was generated with a 0.06 contour level.

There are cases in which the autobuilt model contains junk fragments which were built in places outside the volume of interest. This happens when the input maps for *Buccaneer* are not masked or consist of a large amount of noise. One example is, again, the polycystin 2-l1 data (EMD-8912). The deposited map contains a lot of peripheral noise. *Buccaneer* can mis­interpret these noise peaks, which may carry some resemblance to C^α^ features, as C^α^ atoms. The autobuilt model obtained from the first run through the pipeline contains junk fragments that fill the whole bounding box of the map. After masking the deposited map with the mask map provided, the autobuilt model obtained is better, with no junk fragments built onto noise, and is shown as a model in blue in Fig. 10 ▸. However, the completeness of the model built is still very low owing to the quality of the map.

**Figure 11:**
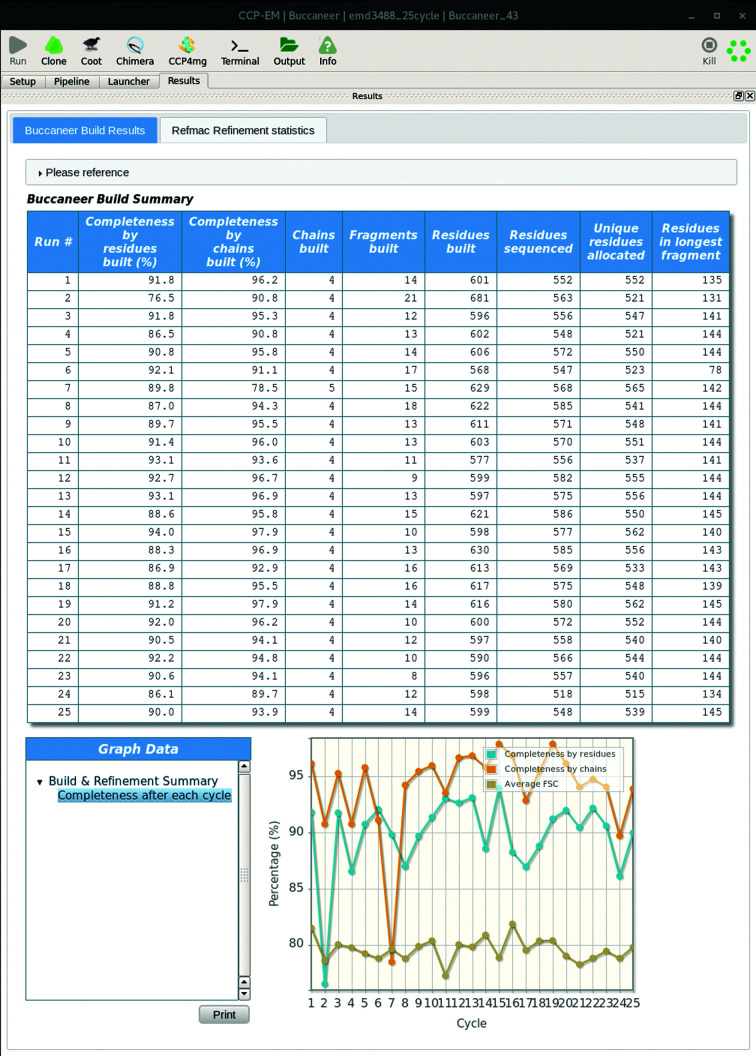
An example of the *Buccaneer* results tab from the *CCP-EM* GUI. Tabulated statistics reported by *Buccaneer* are shown with a graph showing the estimated model completeness with average FSC values for each pipeline cycle.

An automated pipeline has been developed to assist users in the *de novo* building of a model. However, users are still required to inspect and likely to perform some manual editing in order to obtain a high-quality complete model. The results viewer will assist users in selecting the best model to use for further manual editing. In the *Buccaneer* results tab, there is a table with model completeness statistics and a plot with model completeness percentage with average FSC values calculated by *REFMAC*5 (see Fig. 11 ▸). The selected model can be inspected via a molecular-graphics program such as *Coot* (Emsley *et al.*, 2010 ▸), where users can also perform manual deletion, extension and rebuilding of residues (Emsley, 2019*a* ▸,*b* ▸,*c* ▸). Tools within *Coot* such as density fit, Ramachandran plot and geometry analysis are also useful for model validation.

Manually corrected models which need further extension through the automated model-building pipeline can be provided as a starting model. This can be performed by specifying the coordinates file of the model in the ‘Extend model’ box. The keyword ‘nonprotein-radius’ followed by a value for the radius can be given in the ‘Extended options > Keywords’ text box to preserve everything that is neither protein nor water. On the other hand, the keyword ‘known-structure’ can be given to preserve specific chains or atoms from the specified input model to be extended. A radius can also be specified to prevent the building of main-chain atoms within the given radius of the specified atoms. The general syntax for both keywords are as follows:
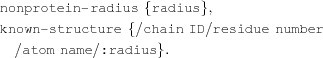



Multiple instructions for ‘known-structure’ can be given using multiple lines of the keyword with various specifications. These keywords will be added as menu options in a future release to ease their usage.

## 4. Conclusions   

Overall, the adjustments made to *Buccaneer* and the pipeline settings in the *CCP-EM* software suite have improved the results from building models into EM maps. Evident improvements in the sequencing step are seen by adapting EM reference structure data in *Buccaneer*. In general, the performance of *Buccaneer* in interpreting EM maps decreases as the overall map resolution becomes worse. However, the overall map resolution reported does not necessarily reflect the quality of the map in all regions. An accurately traced fragment can reduce the length required to achieve a success rate of 80% in sequencing by a few residues. More work needs to be performed in order to improve the model-building algorithm in *Buccaneer* for EM maps. A similar approach will be applied to the *Nautilus* model-building software for nucleic acids (Cowtan, 2014 ▸). However, more than this might be required for *Nautilus* to work properly with EM data, as it uses a different algorithm. The overall effectiveness in detecting the ‘fingerprint’ of nucleic acid bases and the sequencing algorithm requires improvement for it to provide better results.

One point to take note of is that there are many paths to building an atomic model into a volume produced by cryo-EM. As mentioned by Burnley *et al.* (2017 ▸), users can dock models into the cryo-EM volume if a suitable structure or domain is available. Combining different approaches to solve a model is recommended where possible and is helpful when *de novo* model building is challenging, in particular in regions of low resolution.

We have shown in this work that the *de novo* model-building pipeline for EM maps through *CCP-EM* is effective and simple to use. The results obtained also show that it is able to successfully provide at least a good initial model in most cases, especially at better resolutions. Therefore, we hope that this model-building pipeline in *CCP-EM* will benefit users with different experience.

## Supplementary Material

Supplementary Tables. DOI: 10.1107/S2059798320005513/id5008sup1.xlsx

## Figures and Tables

**Figure 9:**
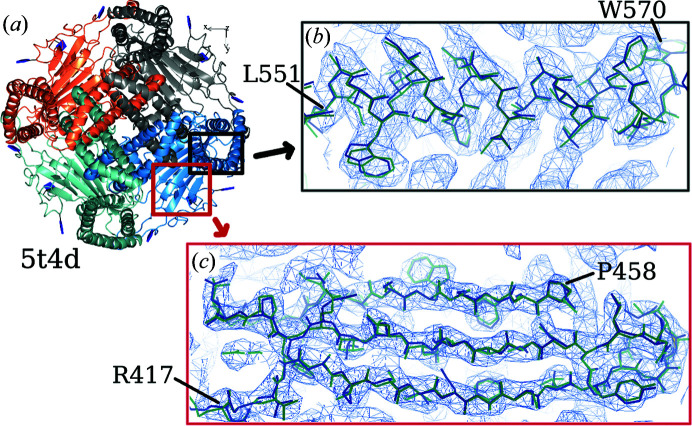
Comparing the deposited model (PDB entry 5t4d) and the autobuilt model built into a map from EMD-8354. (*a*) Overall view of the deposited model. Close-up views are shown of (*B*) a helix chain and (*c*) a β-strand region from the map. The contour level used is 0.05.

**Table 1:** Mean percentage of C^α^ atoms built and residues sequenced correctly (*n* = 208) using various combinations of correlation and fast modes in *Buccaneer*

Mode combination	Mean C^α^ atoms built (%)	Mean residues sequenced (%)
Correlation and fast modes on	41	24
Fast mode on	15	7
Correlation mode on	37	18
Correlation and fast modes off	15	7

**Table 2:** Number of cases that have an up to or greater than 20% improvement in C^α^ atoms built, 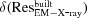, and residues sequenced, 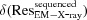, by comparing results from using EM and X-ray reference data in *Buccaneer* (*n* = 208)

	No. of cases (*n* = 208)	
	0% < *x* ≤ 20%	*x* > 20%
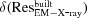	166	3
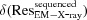	139	41

**Table 3:** Number of cases within different percentage groups of model completeness

	No. of cases (*n* = 208)		
Model completeness criteria (reference data)	<50%	50% ≤ *x* < 75%	≥75%
C^α^ atoms built (EM)	99	79	30
Residues sequenced (EM)	141	47	20
C^α^ atoms built (X-ray)	120	67	21
Residues sequenced (X-ray)	174	25	9
